# Accuracy of computer-assisted implant placement with insertion templates

**DOI:** 10.3205/iprs000094

**Published:** 2016-05-13

**Authors:** Eleni Naziri, Alexander Schramm, Frank Wilde

**Affiliations:** 1 Department of Oral and Plastic Maxillofacial Surgery, Military Hospital Ulm, Germany; 2 Department of Oral and Maxillofacial Surgery, University Hospital Ulm, Germany

**Keywords:** dental implant planning, surgical guides, surgical templates, computer-assisted surgery, computer-aided surgery, guided implant surgery

## Abstract

**Objectives: **The purpose of this study was to assess the accuracy of computer-assisted implant insertion based on computed tomography and template-guided implant placement.

**Material and methods: **A total of 246 implants were placed with the aid of 3D-based transfer templates in 181 consecutive partially edentulous patients. Five groups were formed on the basis of different implant systems, surgical protocols and guide sleeves. After virtual implant planning with the CoDiagnostiX Software, surgical guides were fabricated in a dental laboratory. After implant insertion, the actual implant position was registered intraoperatively and transferred to a model cast. Deviations between the preoperative plan and postoperative implant position were measured in a follow-up computed tomography of the patient’s model casts and image fusion with the preoperative computed tomography.

**Results: **The median deviation between preoperative plan and postoperative implant position was 1.0 mm at the implant shoulder and 1.4 mm at the implant apex. The median angular deviation was 3.6º. There were significantly smaller angular deviations (P=0.000) and significantly lower deviations at the apex (P=0.008) in implants placed for a single-tooth restoration than in those placed at a free-end dental arch. The location of the implant, whether in the upper or lower jaw, did not significantly affect deviations. Increasing implant length had a significant negative influence on deviations from the planned implant position. There was only one significant difference between two out of the five implant systems used.

**Conclusion: **The data of this clinical study demonstrate the accuracy and predictable implant placement when using laboratory-fabricated surgical guides based on computed tomography.

## Zusammenfassung

**Zielsetzung: **Ziel der vorliegenden klinischen Untersuchung war die Evaluation der klinischen Genauigkeit schablonengestützter Implantationen mit laborgefertigten Bohrschablonen auf Basis dreidimensionaler Bildgebung.

**Material und Methoden: **Es wurden insgesamt 246 Implantate mit laborgefertigten Bohrschablonen auf Basis einer dreidimensionaler Bildgebung in 181 teilbezahnten Patienten inseriert. Die inserierten Implantate wurden in Abhängigkeit vom verwendeten Implantatsystem, dem chirurgischen Protokoll und den verwendeten Hülsen in fünf unterschiedliche Gruppen eingeteilt. Nach dreidimensionaler Implantatplanung mit der Software CoDiagnostiX wurden im zahntechnischen Labor Bohrschablonen hergestellt. Nach Implantatinsertion wurde die aktuelle Implantatposition intraoperativ registriert und auf ein Gipsmodell des Patienten übertagen. Durch die Bildfusion der präoperativen Implantatplanung mit der dreidimensionalen Bildgebung des Gipsmodells konnten die Abweichungen zwischen geplanter und erzielter Implantatposition berechnet werden.

**Ergebnisse: **Der Median der Abweichungen zwischen geplanter und erreichter Implantatposition war 1 mm an der Implantatbasis und 1,4 mm an der Implantatspitze. Der Median der Achsenabweichung lag bei 3,6º. Implantate, welche in einer Schaltlücke gesetzt worden waren, zeigten statistisch signifikant kleinere Achsenabweichungen (P=0.000) und geringere Abweichungen an der Implantatspitze (P=0.008) im Vergleich zu Implantaten, welche in einer Freiendsituation inseriert wurden. Es zeigte sich keine Abhängigkeit der Genauigkeit von einer Implantation im Oberkiefer oder Unterkiefer. Die zunehmende Implantatlänge wirkte sich negativ auf die Genauigkeit der erreichten Implantatposition auf. In Abhängigkeit vom Implantatsystem ergab sich ein statistisch signifikanter Unterschied ausschließlich zwischen zwei Systemen.

**Schlussfolgerung: **Die vorliegende klinische Untersuchung konnte die hohe Präzision und Verlässlichkeit einer schablonengeführten Implantatinsertion nach computerassistierter Planung belegen.

## Introduction

In recent years, insertion of dental implants has become a considerably more widespread method in dentistry for achieving functionally and esthetically satisfying results.

The clinical success of implant placement is based on precise preoperative planning. Computer-aided surgery (CAS) techniques are proposed for achieving precise implant positioning and preventing injury of important anatomical structures such as the mandibular nerve or the maxillary sinus [1]. Preoperative planning involves study casts, wax-ups, panoramic X-rays and computed tomography or cone beam computed tomography scans [2]. 3D-based pre-operative diagnosis allows detailed evaluation of the alveolar bone in all three dimensions and facilitates the determination of the optimal number and position of dental implants.

Apart from software for virtual planning, a satisfying method to transfer the preoperative plan to the surgical site is essential. Different transfer techniques are described in literature [3], [4]. Navigation systems can provide three-dimensional orientation, useful information about instrument position and trajectories can be displayed on a monitor in real time within a patient’s 3D imaging data set [5], [6], [7], [8], [9], [10], [11]. In addition to dynamic navigation, mechanical transfer techniques in the form of surgical templates are also available and require less investment in logistics and equipment. In general, there are two ways to manufacture surgical guides. The first option is to fabricate the template manually on the patient’s dental cast. The second is to use rapid prototyping technology following computer-aided design. 

The initial step for both options is to acquire a 3D data set. We recommend that the patient wear a radiological template during the CT or CBCT scan to visualize the ideal subsequent prosthodontic superstructure in accordance with the principle of backward planning. After scanning, data are imported into the planning software and virtual implant planning can be performed. On the basis of these planning data, the surgical template can be manufactured manually using the patient’s dental cast or via CAD/CAM technology (computer-aided design/computer-aided manufacturing). The result of both options is a surgical template in the form of a drilling and implant insertion guide which represents the link between virtual planning and surgical reality.

The purpose of this study was to assess the reliability of implant placement using templates fabricated on dental casts based on computer-assisted implant planning. Therefore, the deviations between preoperative and postoperative implant position were measured at the implant shoulder and the implant apex. The angular deviation from the planned implant axis was also assessed. 

Furthermore, the study describes a reliable technique to evaluate the accuracy of implant placement without additional postoperative patient radiation exposure. 

## Material and methods

This prospective study investigates the template-guided placement of 246 implants in 181 patients (13 women and 168 men) following preoperative computer planning of implant position, length and diameter. The mean age of the patients was 38.1 years. Partially edentulous patients who had given their consent to participate in the study were included. Fully edentulous patients were not enrolled in the study.

Three different types of implants were used:

- Astra Tech Osseospeed (Dentsply Implants, Mölndal, Sweden)
- Straumann ITI Bone Level (Straumann AG, Basel, Switzerland)
- Camlog Promote Plus (Camlog Biotechnologies AG, Basel, Switzerland)

### Study groups

The 246 inserted implants were divided into five groups:

Group I = Partially guided placement of Astra implants

Group II = Fully guided placement of Astra implants

Group III = Camlog Guide System

Group IV = Straumann Guided Surgery System 

Group V = Straumann Steco 

In every group, an implant-specific drilling protocol and appropriate sleeves were used for template-guided implant placement. 

#### Groups I and II

Each of the two groups comprised 50 Astra Tech Osseospeed implants (Dentsply Implants, Sweden) that were placed either in a partially guided (Group I) or fully guided (Group II) manner. In both groups, implants with a diameter of 3.5 mm, 4.0 mm and 5.0 mm were used.

#### Group I

**Figure 1:**
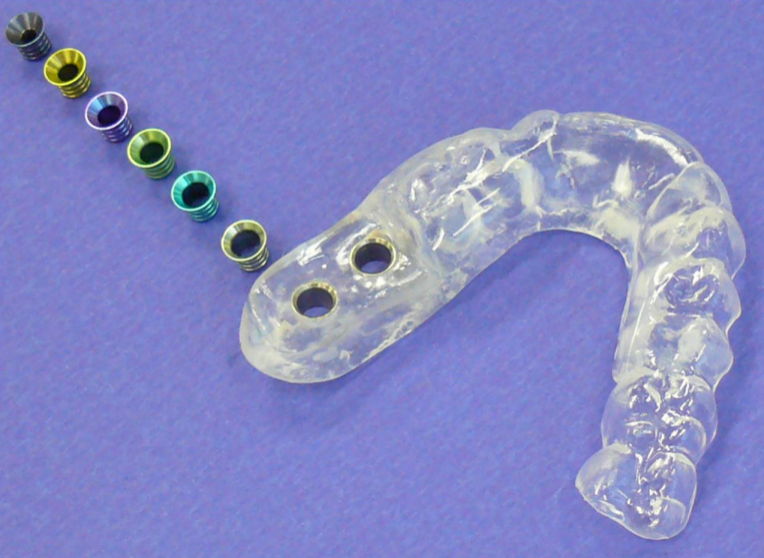
Steco sleeve system for template-guided implant placement. The sleeves, which are color-coded and have different diameters, fit precisely into the guide sleeves of the template.

In this group, in which Astra implants were inserted in a partially guided fashion, only the first two twist drills were precisely guided using replaceable Steco titanium sleeves (steco-system-technik GmbH & Co.KG, Hamburg, Germany) (Figure 1 (Fig. 1)). The guided twist drills had a diameter of 2.0 mm or 3.2 mm. In this group, implants with a diameter of 4.0 mm were inserted in a guided fashion using the guide sleeve of the template. Implants with a diameter of 3.5 mm or 5.0 mm were placed freehand without the aid of a sleeve. Whereas implants with a diameter of 3.5 mm were inserted into the jaw through the guide sleeve of the template in an approximative manner, implants with a diameter of 5.0 mm were placed completely freehand following the guided preparation of the implant site. 

#### Group II

In this group, in which Astra implants were inserted in a fully guided fashion, sleeve-guided preparation was possible for holes with a maximum diameter of 3.7 mm using guided twist drills with a diameter of 2.0 mm, 3.2 mm or 3.7 mm. Implants with a diameter of 3.5 mm or 4.0 mm were inserted in a precise manner using appropriate Steco titanium sleeves. As in Group I, implants with a diameter of 5.0 mm were placed completely freehand following the guided preparation of holes with a maximum diameter of 3.7 mm.

#### Group III

**Figure 2:**
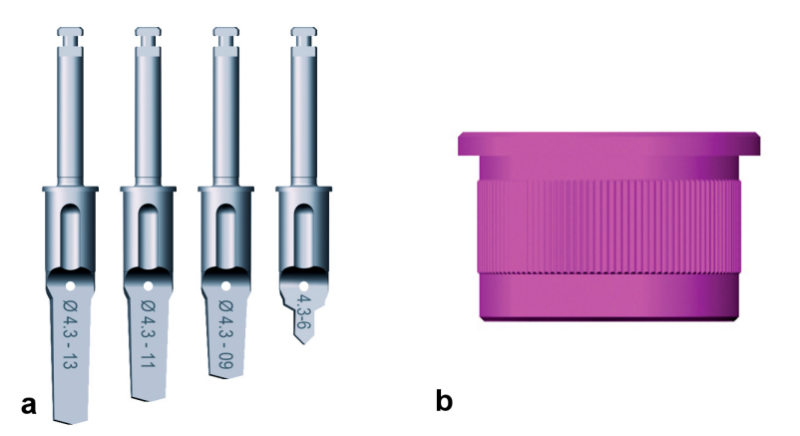
Camlog Guide System a) Camlog Guide System drills of increasing length and a diameter of 4.3 mm. b) Camlog guide sleeve. Drilling and implant placement were carried out through one and the same guide sleeve integrated into the template.

The Camlog Guide System was used in this group. A total of 50 Camlog Promote Plus implants (Camlog Biotechnologies AG, Switzerland) with a diameter of 3.8 mm or 4.3 mm were inserted. Drills of increasing length were used for the guided preparation of the implant site. All drills had a diameter corresponding to that of the planned implant (Figure 2a (Fig. 2)). Drilling and implant placement were carried out through a guide sleeve integrated into the template (Figure 2b (Fig. 2)).

#### Group IV

**Figure 3:**
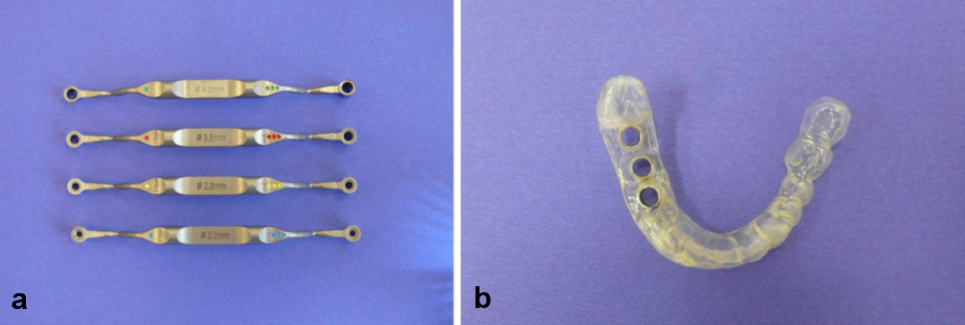
Straumann Guided Surgery System a) The four color-coded drill handles of the Straumann Guided Surgery System. Each of the four drill handles features a cylinder with an additional height of 1 mm at the left end and 3 mm at the right end. b) The drill handles fit precisely into the sleeves that are integrated into the template.

In this group, the Straumann Guided Surgery System was used. This system includes four special drill handles, which accurately fit into sleeves that are integrated into the template. Each of the four drill handles features a cylinder with an additional height of 1 mm at one end and 3 mm at the other. Together with the length of the drill and the template with the guide sleeves, these drill handles and cylinders allow the planned length of the implant to be transferred to the surgical site. For every drilling step (2.2 mm, 2.8 mm, 3.5 mm, and 4.2 mm), drills of three different lengths (16 mm, 20 mm and 24 mm) with depth stops are available. The surgical protocol specifies which drill and which drill handle cylinder should be used for the various implants. Milling cutters with three different diameters (2.8 mm, 3.5 mm and 4.2 mm) are used in order to prepare the alveolar ridge and obtain a flat bone surface. When a guide sleeve with an internal diameter of 5.0 mm is used, the Straumann Guided Surgery System allows the final drilling for implants with a diameter of 4.8 mm to be performed in a template-guided manner. Apart from implant placement, thread cutting and profile drilling can be performed in a template-guided fashion in order to meet specific implant requirements. The insertion of an implant with a diameter of 4.8 mm, however, is guided only by the guide sleeve without the use of drill handles (Figure 3 (Fig. 3)).

#### Group V

**Figure 4:**
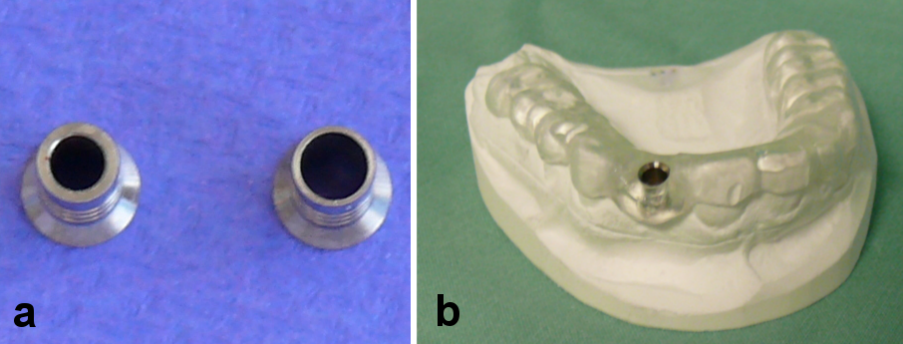
Straumann Steco system a) Steco sleeves for guiding the first two preparation steps; b) Drill guide with an integrated guide sleeve

The Straumann Steco group comprised 46 implants. Only the first two preparation steps were performed in a template-guided manner. For this purpose, drills with a diameter of 2.2 mm or 2.8 mm and Steco sleeves that were inserted into the guide sleeve were used (Figure 4a (Fig. 4)). Further drilling including thread cutting and profile drilling as well as implant placement were performed in an approximative manner using the guide sleeve with an internal diameter of 5 mm (Figure 4b (Fig. 4)).

### Implant planning

**Figure 5:**
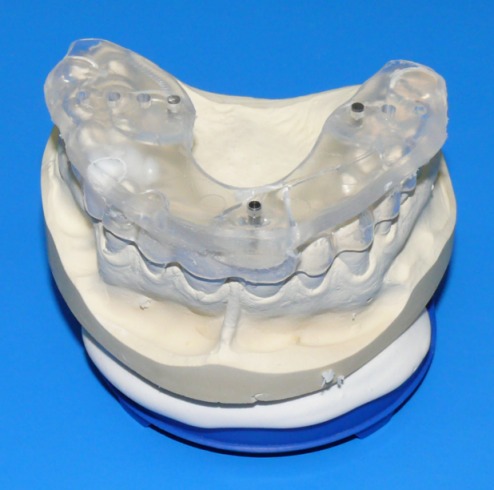
Scan template after the preparation of a wax-up and set-up of the planned prosthetic restoration. Three titanium pins were attached lingual to the tooth row by polymerization.

Following a wax-up and set-up of the planned prosthetic restoration, a scan template was created for every patient in accordance with the principle of backwards planning, which starts with study casts. Three titanium pins were inserted into the scan templates lingual to the tooth row for the spatial referencing of image data and image fusion (Figure 5 (Fig. 5)). 

Computed tomography (CT) was performed in order to acquire three-dimensional image data sets for computer planning (Somatom Definition Siemens, Erlangen, Germany). The following scanner settings were used: tube potential 120 kV, tube current 230 mAs, increment 0.4 mm, rotation time 1s, collimation 0.6 mm. CT data sets were reconstructed at a slice thickness of 1 mm. 

**Figure 6:**
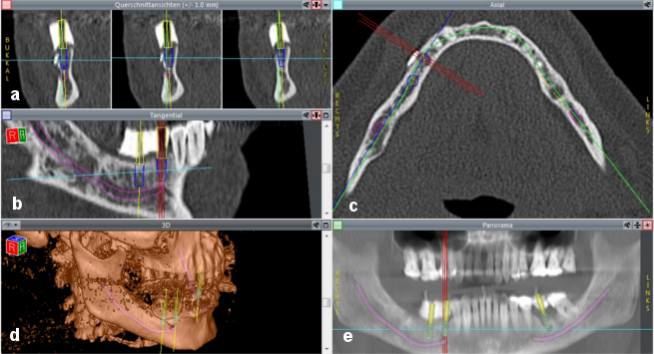
Screenshot demonstrating implant planning a) coronal view; b) sagittal view; c) axial view; d) 3D view; e) pseudo-panoramic radiograph. The purple line demonstrates the inferior alveolar nerve. The blue cylinders represent the implants selected from the implant library. The yellow cylinders show the virtual abutments, which allow the implants to be placed in relation to the planned positions of the abutments. This allows an adequate prosthetic restoration to be achieved on the basis of the principle of backwards planning. Once the set-up was completed, the scan template was filled with barium sulfate. The planned location of the abutments was created in the template with a pilot drill. The green line (c) represents the panoramic curve, which allows a pseudo-panoramic radiograph to be generated. The yellow lines (a, b and d) show the vertical axis of the implant. The light blue lines show the horizontal axis of the implant. The three red lines (b, c and e) represent the axes of the implant that is being positioned.

Implant planning and simulation were performed with CoDiagnostiX software (version 6.0, IVS Solutions AG, Chemnitz, Germany). CT data (in DICOM format) were imported into the software, which was then used to virtually place implants into their position and to assess them in multiplanar (axial, coronal and sagittal) and three-dimensional views. In addition, a pseudo-panoramic radiograph was created (Figure 6a-e (Fig. 6)).

A special software tool was used to identify and virtually visualize the inferior alveolar nerve. The software features an automatic warning function in order to prevent implants from being positioned too close to the nerve during planning. 

For virtual implant positioning, appropriate implants can be selected from an implant database and exchanged as often as required. This database contains a wide variety of implants from major international implant manufacturers. The implants are grouped according to available lengths and diameters.

Once an implant has been selected, it can be positioned in the image data set in all possible views.

In addition, an abutment can be freely defined and virtually assigned to an implant. Abutment diameter, height, inclination and rotation can be changed as required. A virtual abutment allows the ideal implant position to be determined depending on the requirements that the implant axes must meet for an appropriate prosthetic restoration. Using the backwards planning approach, it is thus possible to achieve an esthetically and functionally satisfying prosthetic restoration. The procedure benefits from the use of barium sulfate that is filled into the scan template once the set-up has been completed. A pilot drill is then used to create the planned location of the abutments in the template. Implants can thus be positioned accurately in three dimensions on the basis of the planned prosthetic restoration (Figure 6a,b,e (Fig. 6)).

### Transfer of virtual planning to the surgical site

**Figure 7:**
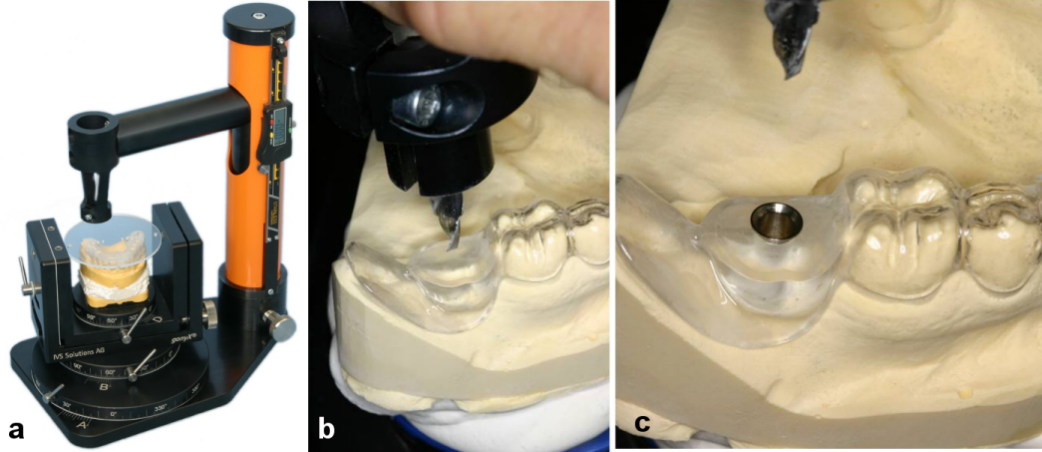
Creation of drill guides using a gonyX table (IVS Solutions AG, Chemnitz, Germany) on the basis of virtual planning a) Transfer of computer-assisted implant planning to the implantation template using the gonyX table; b) Drilling into the template; c) Template with guide sleeve

Following virtual implant planning and referencing, data were transferred to a dental laboratory. Based on virtual planning, drill guides were created using a gonyX table (IVS Solutions AG, Chemnitz, Germany) in the laboratory (Figure 7a-c (Fig. 7)).

After the templates had been produced and the exact drilling protocol had been printed, the preparation of the implant site and the placement of the implant were performed in a template-guided manner. Following the administration of local anesthetics for pain relief, a flap was raised using a crestal incision and the drill guide was positioned appropriately. The implant bed was prepared in accordance with the manufacturer's instructions. In all cases, the alveolar bone was exposed following flap elevation. Flapless surgery was deliberately avoided.

**Figure 8:**
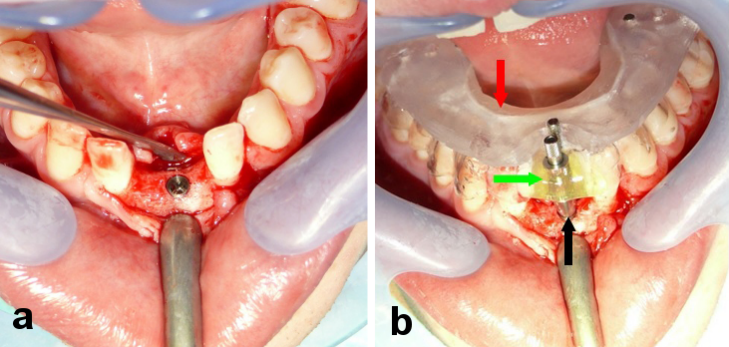
a) Intraoperative view following flap elevation and implant placement; b) The impression post that had been placed into the implant (black arrow) was attached to the reworked scan template (red arrow) by polymerization (green arrow) using a light-curing material (Triad Gel, Dentsply, York, United States).

Once the implant had been placed, the achieved implant position was registered during the surgical procedure as follows. Parallel to the fabrication of the drill guide, the scan template that had been used for obtaining image data was reworked into a transfer template by removing the buccal portion of the template in the region of the implant. During this process, care was taken to protect the titanium reference pins. Following implant insertion, standardized impression posts were inserted into the implants. Following the intraoperative placement of the reworked scan template, the impression posts were attached to the template by polymerization using a light-curing material (Triad Gel, Dentsply, York, United States) (Figure 8 (Fig. 8)).

The former scan template thus allowed the position of the implant to be registered during the surgical procedure. 

### Transfer of the surgical results to the study cast

**Figure 9:**
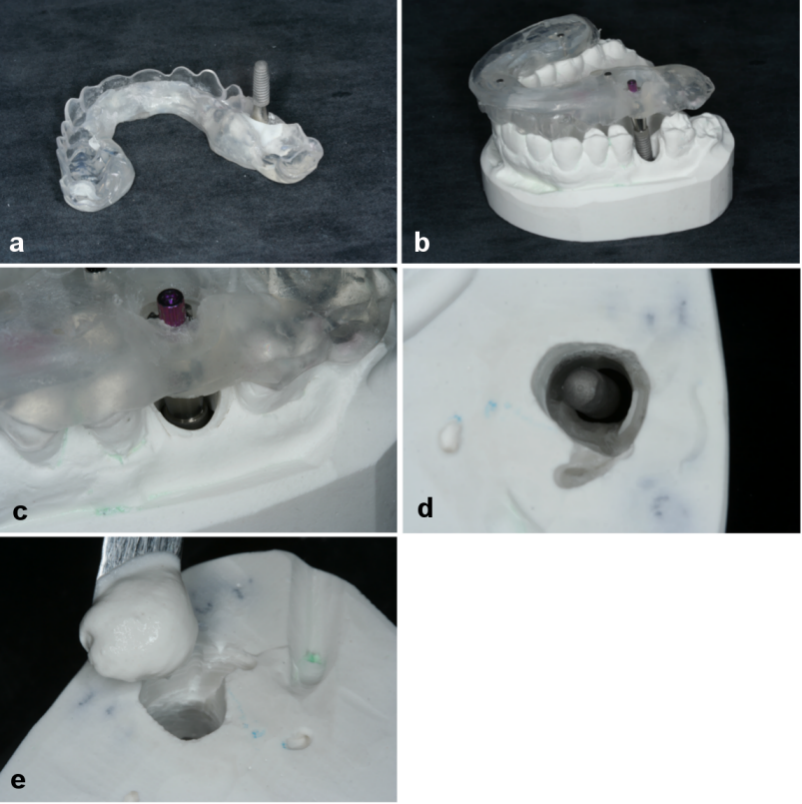
Transfer of the surgical results to the study cast a) The implant analogue was screwed onto the impression post. b) and c) The scan template with the impression post was inserted into the cast. d) The cast with the implant; e) The implant is getting fixed with plaster.

In the dental laboratory, an implant analogue with the same dimensions as the clinically placed implant was used to transfer the actual implant position to the patient-specific study cast. Using the impression post that had been attached to the template by polymerization, the implant analogue was repositioned and fixed. Following the removal of material in the region of the implant, the template with the implant analogue was placed accurately on the remaining teeth. The implant analogue was fixed into the cast with plaster and reflected the patient situation (Figure 9 (Fig. 9)).

### Evaluation 

New CT scans were obtained from every cast simulating the postoperative implant position and the associated scan template. The same scanner settings that had been selected for implant planning were used again. Three-dimensional radiographic images of postoperative implant positions were thus obtained without additional patient radiation exposure. The impression posts that been attached to the templates during the surgical procedure were removed in order not to cause artifacts. 

In a next step, the Voxim skeleton module (IVS Solutions AG, Chemnitz, Germany) was used to localize and mark the center of the cranial surface of each reference pin on preoperative and postoperative CT scans in order to allow the postoperative situation to be superimposed onto preoperative planning.

**Figure 10:**
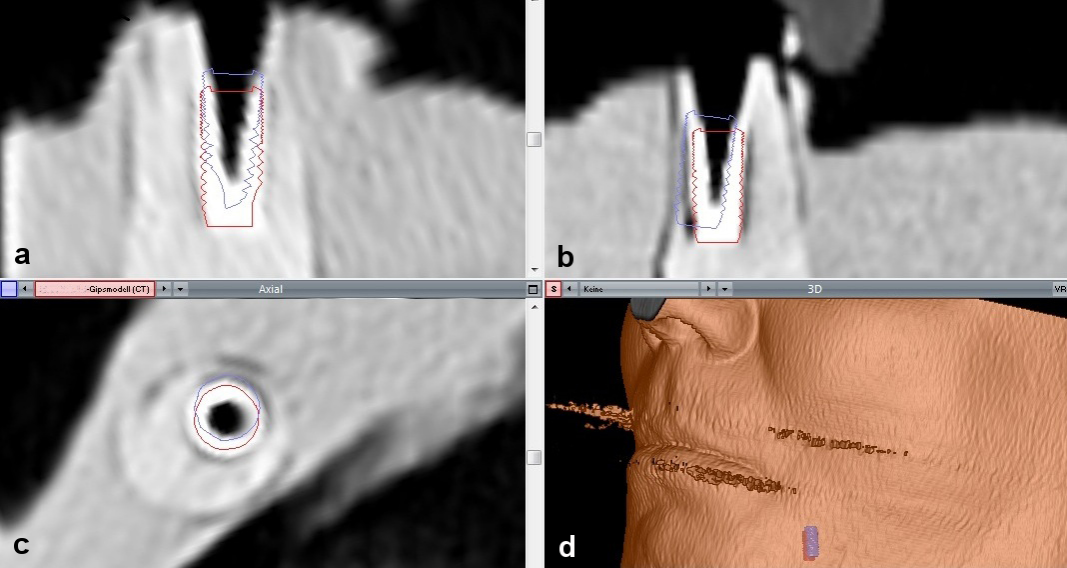
Screenshot after automatic image fusion of preoperative planning and a postoperative CT of the cast with an implant analogue (Voxim skeleton module, IVS Solutions AG, Chemnitz, Germany) a) saggital view; b) coronal view; c) axial view; e) 3D The virtual implant that was manually fused with the implant shown on the CT scan is represented by red contour lines. These red lines show the actual position of the implant. The blue contour lines show the planned position of the implant. The figure presents a case with deviations above average for illustration reasons.

Following software-based automatic image fusion, a virtual implant with the same dimensions as the clinically placed implant was positioned manually on the implant shown on the CT scan. This implant was highlighted by red contour lines. An assessment of implant position was possible in all planes. The originally planned implant was shown by blue contour lines (Figure 10 (Fig. 10)).

**Figure 11:**
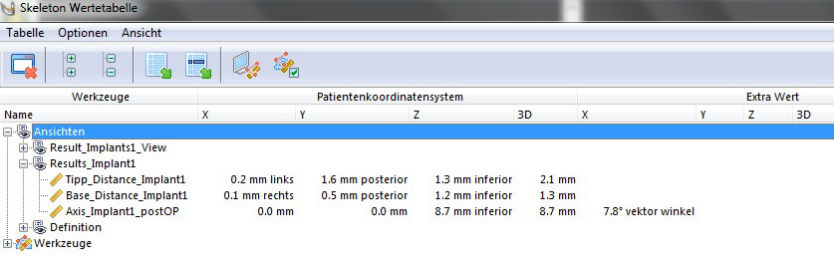
Screenshot of the results that were automatically produced by the skeleton module of Voxim. The table shows the deviations in the x, y and z axes, 3D deviations and implant axis deviations (in degrees) between actual and planned implant positions.

The skeleton module of Voxim allowed deviations between the planned and postoperative implant positions to be automatically detected along the x, y and z axes (Figure 11 (Fig. 11)).

The z axis is defined by the axis of the planned implant and describes deviations in the superior-inferior direction. The y axis is perpendicular to the z axis. Accordingly, the y axis shows horizontal deviations in implant position in the oro-vestibular direction. The x axis is perpendicular to the other two axes and shows horizontal deviations in the mesio-distal direction. These deviations were assessed by the software both at the level of the implant shoulder and at the level of the implant apex. The total deviation in space (3D deviation), which corresponds to the Euclidean distance, and the deviation between the axes of the planned and actual implants were determined as well.

### Statistical analysis

Descriptive data analysis was completed using the software SPSS, version 19. The Kolmogorov-Smirnov test and the Shapiro-Wilk test revealed that the distribution of the data was not homogeneous. Therefore, we analyzed the data using the median, maximum, minimum, range and interquartile range. The relation between the preoperative planning data and the actual data recorded intraoperatively was visualized with boxplots. A Kruskal-Wallis test was performed to calculate statistical significances. Values of p≤0.05 were considered significant and values of p≤0.005 were considered highly significant. 

## Results

### Overall evaluation

In total, 246 implants were placed without further complications. No implant loss was recorded in the early healing period of the three first months. A total of 236 implants could be included in the statistical analysis. The remaining 10 implants were considered dropouts. A total of 98 implants were placed in the maxilla and 138 implants were placed in the mandible. The majority of implants were placed in edentulous gaps for further single-tooth restorations (66%), while the rest were implants placed in a free-end dental arch (34%). 

In 12 of the 236 implants, there was a discrepancy between actual length and preoperative planning. This means that in 94.9% of cases, the actual length of the implant was as planned. 

Eight implants were one level and two implants were two levels shorter than originally planned owing to the risk of perforation of the maxillary sinus or the mandibular nerve canal. Two implants were placed with one size up from the length that was originally planned in order to ensure sufficient primary stability. As a result of this, the Z value (vertical deviation) and 3D value (Euclidean distance) were considered partial dropouts in these 12 implants and were not included in the statistical analysis. The Z value, 3D value and the X and Y value at the implant apex were also considered partial dropouts in another two cases where the implants were placed 3 mm deeper than planned.

Of the 236 implants, 230 (97.5%) were placed with the planned diameter. A larger diameter was used in two cases, as bone availability had been underestimated prior to surgery or because of a lack of primary stability. In six cases the bone situation indicated a thinner implant than planned.

A total of ten implants were considered total dropouts. In six of these cases, drilling steps were performed without templates because inadequate mouth opening meant that drilling with the templates was not possible or because of an anatomically inappropriate pilot drilling. In another two cases, the implants had to be inserted without a template. In one case, the intraoperative impression was inaccurate. In the tenth case the implant pick-up for the intraoperative impression could not be properly placed and left a 2 mm gap to the implant shoulder. 

The median of horizontal deviations between planned and achieved implant position measured in mesial-distal direction was 0.3 mm at the implant shoulder and 0.7 mm at the implant apex, with maximum deviations of 2.6 mm at the implant shoulder and 3.3 mm at the implant apex (Table 1 (Tab. 1)). 

The median of horizontal deviations in oro-vestibular direction was 0.3 mm at the implant shoulder and 0.6 mm at the implant apex, with maximum deviations of 2.7 mm at the implant shoulder and 3.8 mm at the implant apex.

Along the longitudinal axis of the implant (Z axis), the median deviation both at the shoulder and the apex was 0.6 mm, with a maximum deviation of 4 mm.

The median of deviations in space (3D value) was 1 mm at the implant shoulder and 1.4 mm at the implant apex, with maximum deviations of 4.3 mm at the implant shoulder and 5.5 mm at the implant apex. 

**Figure 12:**
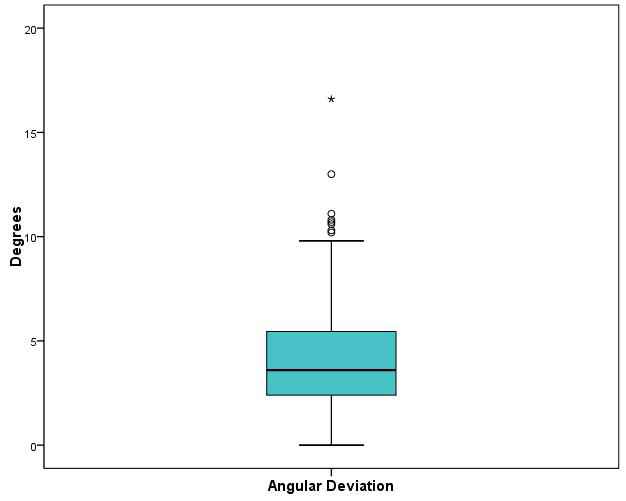
Boxplot of the angular deviations in degrees

When it came to angular deviation, 50% of deviations were between 2.4º und 5.46º, with a median of 3.6º and maximum of 16.6º (Figure 12 (Fig. 12)).

### Comparison of implant systems

**Figure 13:**
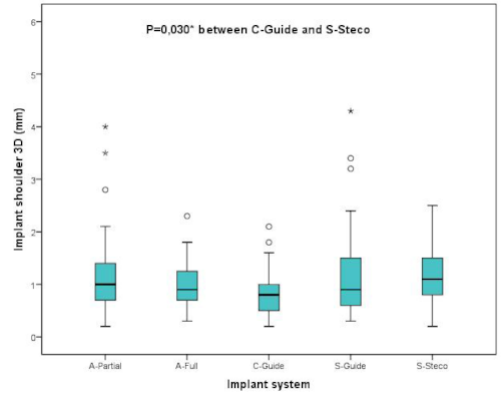
Boxplots of the different implant systems according to the 3D deviations at the implant shoulder in mm

A comparison of the results achieved with the different implant systems showed a statistically significant difference between the implant systems Straumann Steco and Camlog Guide at the implant shoulder in 3D (p≤0.030). As far as deviations in space (3D value) were concerned, Camlog Guide, with a median deviation of 0.8 mm, was significantly more precise than Straumann Steco, with a median of 1.1 mm. However, no additional statistically significant deviations were revealed by comparing the different systems tested in this study (Figure 13 (Fig. 13)).

### Comparison of clinical indications

**Figure 14:**
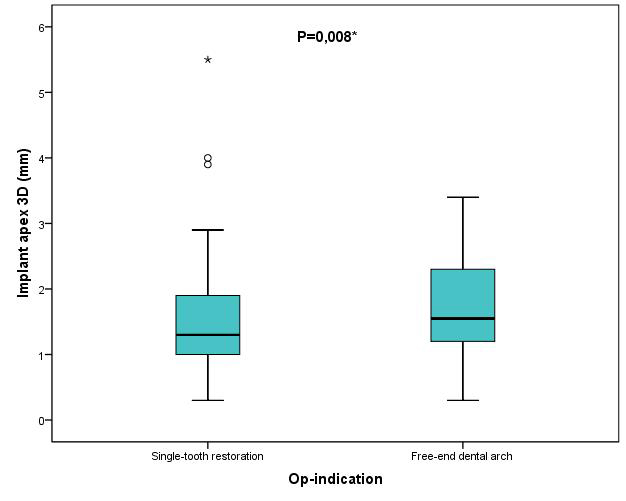
Boxplots of the different indications (single-tooth restoration or free-end dental arch) according to the 3D deviations at the implant apex in mm

**Figure 15:**
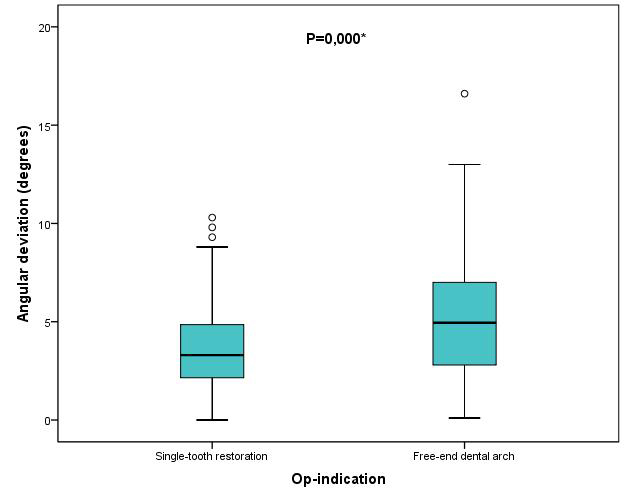
Boxplots of the different op-indications (single-tooth restoration or free-end dental arch) according to the angular deviations in degrees

Results were also analyzed with regard to the different clinical indications. A total of 156 implants were placed to provide a single-tooth restoration in interdental gaps. Another 80 implants were placed in a free-end dental arch. Implant placement for restoration of a single interdental gap yielded statistically significantly better results than placement of an implant in a free-end dental arch. Implant positioning for a single-tooth restoration was significantly more precise at the implant apex regarding deviations in space (p≤0.008) and angular deviation (p≤0.000) than implant positioning in a free-end dental arch (Figure 14 (Fig. 14), Figure 15 (Fig. 15)). The median of 3D deviations at the implant apex and angular deviation was 1.3 mm and 3.3º, respectively, for a single-tooth restoration and 1.55 mm and 4.7º, respectively, for implants placed in a free-end dental arch. No additional statistically significant deviations could be observed by comparing the different clinical indications.

### Comparison of implant length

**Figure 16:**
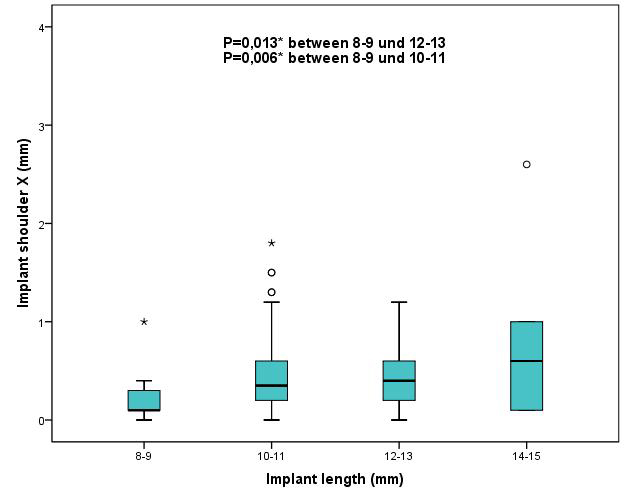
Boxplots of the different implant lengths 8–9 mm, 10–11 mm, 12–13 mm, 14–15 mm according to the deviations at the implant shoulder X (mesial-distal direction) in mm

**Figure 17:**
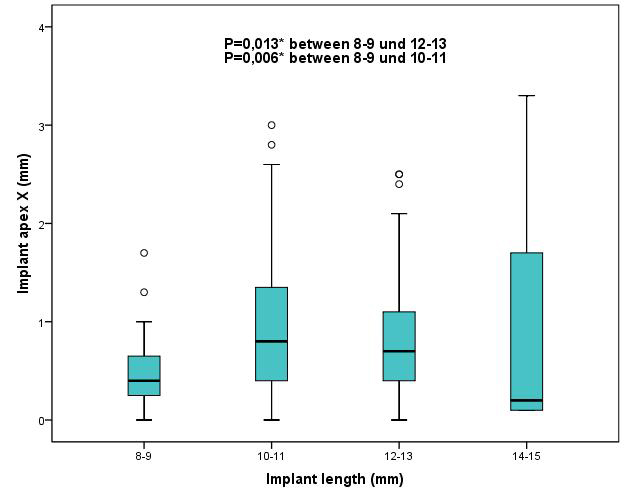
Boxplots of the different implant lengths 8–9 mm, 10–11 mm, 12–13 mm, 14–15 mm according to the deviations at the implant apex X (mesial-distal direction) in mm

Results were also analyzed with regard to implant length in order to determine whether implant length can influence the precision of implant placement. To do so, a total of 236 implants were divided into four groups with implant lengths of 8–9 mm (n=20), 10–11 mm (n=112), 12–13 mm (n= 99) and 14 mm (n=5), respectively. The results revealed that insertion of implants with a length of 8–9 mm was significantly more precise (Figure 16 (Fig. 16), Figure 17 (Fig. 17)) than of those with a length of 10–11 mm and 12–13 mm in mesio-distal direction (X axis) at the implant shoulder (p≤0.006) and implant apex (p≤0.013). No other statistically significant differences could be found between any other results with regard to deviations in the Y axis and Z axis, deviations of the 3D values and angular deviations.

### Comparison of implant sites

The results were also examined in order to determine whether the implant being inserted in the upper or lower jaw had an effect on precision. In the maxilla, implant sites included the upper first incisor (n=9), upper second incisor (n=6), upper first premolar (n=14), upper second premolar (n=26) and upper first molar (n=38). In the mandible, implants were placed as the lower first premolar (n=3), lower second premolar (n=25), lower first molar (n=84) und lower second molar (n=24). The implant sites lower first incisor (n=1) und lower second incisor (n=1) were not included in the statistical analysis because of the small number of cases. However, no statistically significant differences could be found between the implant sites in any computed evaluations.

## Discussion

Planning of dental implant positioning and its accurate transfer to the surgical site can be considered one of the most important factors in successful implant-supported restoration [12], [13]. Appropriate planning software allows surgeons to already integrate the ideal prosthetic restoration in the planning step of dental implant positioning.

The aim of this study was to assess the accuracy of implant positioning when using laboratory-fabricated, 3D-based templates following virtual planning with CoDiagnostiX software (IVS Solutions AG, Chemnitz, Germany). Throughout the study, to the focus was also on avoiding postoperative 3D imaging in order to protect patients from any unnecessary radiation.

The results of this evaluation indicate a high congruence between preoperative planning data and intraoperative results. The planned and actual values were consistent in 100% of cases regarding implant positioning, in 94.9% of cases regarding implant length and in 97.5% of cases regarding implant diameter. These results correspond to those reported by Fortin et al. in 2003 [14] and Behneke et al. in 2009 [15].

In 2003, Fortin et al. [14] described a CT-based computer-aided implant planning approach that uses CAD implant software. Planning data were transferred to the surgical site using an acrylic block with integrated titanium guide tubes. In 30 partially or fully edentulous patients, the congruence between preoperative planning and the actual intraoperative situation was 96.6% with regard to implant position, 98.9% with regard to implant length and 96.8% with regard to implant diameter.

In a 2009 study by Behneke et al. [15], a total of 131 implants were placed using laboratory-fabricated templates after planning with Med3D and CoDiagnostiX Software. Congruence between preoperative planning and the actual intraoperative situation was 97.7% with regard to implant length and 96.2% with regard to implant diameter.

In general, the results of our study demonstrate that it is possible to transfer a virtual implant position based on computer planning to the surgical site precisely. However, deviations were observed and require detailed analysis. The mean deviations in the X and Y axis at the implant shoulder (0.42 mm and 0.43 mm, respectively) and at the implant apex (0.85 mm and 0.72 mm, respectively) were less than 1 mm. This result confirms the empirical requirement of a minimum distance of 1 mm to important anatomical structures (inferior alveolar nerve, adjacent tooth). The maximum deviations in the X and Y axis were 2.6 und 2.7 mm respectively at the implant shoulder and 3.3 and 3.8 mm respectively at the implant apex. The maximum deviations of 3.3 mm and 3.8 mm respectively at the implant apex were found in a case where an implant of 14 mm in length was inserted. Our results proved that increasing implant length has a negative influence on the precision of implant positioning. Therefore, the use of long implants must be carefully considered, especially in cases of implant placement close to the mandibular nerve canal or small edentulous gaps, in order to avoid extreme deviations and protect important anatomical structures. 

In the Z axis, the mean deviation was 0.73 mm, with a maximum deviation of 4 mm. In three of the implant systems (Astra partial guided, Astra full guided and Straumann Steco) drilling was performed without using a stop. Furthermore, the vertical dimension depends on the crestal bone level and esthetic requirements which can be best defined and controlled visually during surgery. For these reasons no vertical safety distance can be recommended and flapless surgery must be avoided. 

The mean angular deviation of the 236 implants placed was less than 5º (4.1º). This leads us to conclude that in most cases there is no need for angled abutments when placing implants with 3D-based insertion guides. This is an advantage for the mechanical load-bearing capacity of the implants and generally for long-term preservation of the peri-implant bone.

Nevertheless, for the prevention of injuries to anatomical structures, maximum deviations are the most important values to consider. The greatest angular deviation observed was 16.6º. This deviation was seen in an implant (4.8 mm in diameter/10 mm in length) that was inserted in a free-end dental arch in combination with an internal sinus elevation in the Straumann Steco group. This significant deviation may be explained by the fact that in the Straumann Steco group only the first two steps of surgical site preparation were performed in a template-guided manner whereas the next drilling steps and the positioning of the implant were done freehand without the template. 

Furthermore our results verified that implantation in a free-end dental arch has a statistically significant negative influence on the precision of implant insertion compared to implantation in an interdental gap. The most likely explanation for this is that the surgical guide is only partially tooth-supported in free-end dental arch implantation. Therefore, we propose that the support of the template is a very important factor in the accuracy of template-based implant placement. A possible solution for this could be the insertion of temporary implants to stabilize the insertion guide, especially when paranerval implantation is planned. However, this would involve considerable effort and may only be recommended in select cases.

The comparison between all implant systems showed a significant difference between the groups Camlog Guide and Straumann Steco in the 3D value at the implant shoulder but there were no other statistically significant differences between the other groups. As previously mentioned, in the Straumann Steco group only the first two steps of surgical site preparation were performed in a template–guided manner whereas the next drilling steps and the positioning of the implant were done freehand without the template. From these data we conclude that fully guided steps of the drilling protocol with special drills and probably a depth stop, as used in the Camlog Guide system, have an advantage with regard to accuracy over systems with only partially guided drilling steps, as in the Straumann Steco group. Nevertheless, it can also be concluded that the Steco sleeve system, if used as a fully guided system, can achieve results that are similarly precise as the fully guided Camlog Guide and the Straumann Guide systems. 

Implant length seems to be also a factor in the accuracy of template-based implant placement. The greater deviations measured in longer implants may be due to the angle of the drill during implant placement. The angle may be affected by the difficulty of inserting longer drills in the templates if mouth opening is limited. As a result, the drill does not pass through the drill sleeve at a right angle, which results in an angle of surgical site preparation that is different from the plan. This interpretation is also supported by the fact that the significant deviations associated with implant length can only be found in the mesio-distal direction. In conclusion, shorter implants with a length of up to 11 mm should be recommended in order to avoid greater deviations during template-based implant placement, especially in close proximity to important anatomical structures. 

Most available publications on the accuracy of surgical transfer using template-supported 3D-based implant planning are in-vitro studies [16], [17], [18], [19], [3]. Only three of them are clinical trials [20], [21], [22]. A few review articles [23], [24], [25] point out that further studies are necessary to determine the reproducibility and precision under clinical circumstances. In a 2008 in-vitro study by Kalt and Gehrke [3], 48 implants were placed into eight study models made from calf ribs. Virtual planning was performed with the med3D Software (med3D GmbH, Heidelberg, Germany) and computed tomography was performed after implant placement with Steco sleeve-guided drilling templates (Steco-System-Technik GmbH, Hamburg, Germany). The study showed that the precision of implant placement decreases with the number of steps for guided surgical site preparation. Our study suggests a similar correlation when we compare the partially guided groups (Astra partial and Straumann Steco) with the fully guided groups (Astra full, Camlog Guide, Straumann Guide). Nevertheless, the deviations measured in the 2008 study by Kalt and Gehrke were slightly smaller, which can be explained by the clinical circumstances of our study, which are not comparable with those in vitro. In other in-vitro studies by Horwitz et al. in 2009 [19] and Barnea et al. in 2010 [16] deviations comparable to our in-vivo study were found at the implant shoulder, the implant apex and with regard to angular deviation.

In one last in-vitro study by Dreiseidler et al. 2009 [17], 54 implant positions were planned for 10 partially edentulous models and placed using surgical guides ordered from the manufacturer (Sicat, Bonn, Germany). The deviations were determined to be smaller than 500 µm and the mean angle deviation was 1.18º. These very precise results are unlike those of any other study, which makes them questionable.

The most important difference between our study and the few clinical studies previously published is that we did not expose the patients to any additional radiation in postoperative CT or CBCT imaging for the purpose of our study. 

In one 2010 in-vivo study by Behneke et al. [20], a total of 132 implants were placed using insertion guides with titanium sleeves after implant planning with med3D software (med3D GmbH, Heidelberg, Germany). Of the 132 implants, 89 were Straumann implants placed with the Straumann Guide System (Straumann AG, Basel, Switzerland) and 43 were Nobel Replace implants placed with the Nobel Guide System (Nobel Biocare AG, Gothenburg, Sweden). A postoperative CBCT was performed to fuse with preoperative planning data in order to calculate deviations. The mean deviations were 0.32 mm at the implant shoulder and 0.49 mm at the implant apex and a mean angular deviation of 2.1°. As such, the results are better than those of our study (1.1 mm at the implant shoulder, 1.54 mm at the implant apex, 4.1° mean angular deviation). It is also remarkable that a second study by Behneke et al. in 2012 [26] found no significant difference between the results of an implant placed for a single-tooth restoration and an implant placed in a free-end dental arch, which does not correspond to the results of our study either. We cannot conclusively explain the remarkably better results of the study by Behneke et al. Our study only differed from theirs in that we used CoDiagnostiX software (IVS Solutions AG, Chemnitz, Germany) for implant planning and other implant and guide systems (except the Straumann Guide System). However, in our study not even the Straumann Guide System could achieve the results of Behneke et al.

In another clinical study by Schlieper in 2010 [22], results similar to ours were published. Schlieper inserted 32 Camlog implants (Camlog, Basel, Switzerland) in 14 patients using a Sicat surgical guide (Sicat, Bonn, Germany) and the Camlog guide single-sleeve system (Camlog, Basel, Switzerland). A postoperative CBCT was also performed after placement of the implants. The mean deviation in the mesial-distal direction was 0.47 mm at the implant shoulder and 0.82 mm at the implant apex (our study: 0.42 mm at the implant shoulder and 0.85 mm at the implant apex) and the mean deviation in the inferior-superior direction was 0.25 mm (our study: 0.73 mm). The mean angular deviation was 3.07º (our study: 4.1º). The results were not examined in the oro-vestibular direction. The implant site was also not found to have a significant influence on the deviations. 

There is only one clinical study in the literature which determines deviations between planned and achieved implant position without an additional 3D image of the patient. In this 2010 study by Nickenig et al. [21], 23 implants were placed in 10 patients after planning with CoDiagnostiX software (IVS Solutions, Chemnitz, Germany) and placing Nobel Biocare implants (Nobel Biocare AB, Gothenburg, Sweden) using tube-in-tube guide sleeves. All implants were placed in a free-end gap situation in the lower jaw and a standard diameter of 4 mm was used. No implant was shorter than 10 mm. Similar to our study, the preoperative situation was fused with a cone-beam CT of the master model including implant replicas of the prosthetic restoration to evaluate the results. The results were comparable to our study, with a mean deviation in the oro-vestibular direction of 0.9 mm at the implant shoulder and 0.9 mm at the implant apex (our study: 0.43 mm at the implant shoulder and 0.72 mm at the implant apex). In the mesial-distal direction, the study by Nickenig et al. showed deviations of 0.9 mm at the implant shoulder and 0.6 mm at the implant apex (our study: 0.42 mm at the implant shoulder and 0.85 mm at the implant apex). The mean angular deviation was 4.2º (our study: 4.1º). 

Our study should also be compared with alternative techniques for transferring implant planning to the surgical site.

Freehand drilling is the most inaccurate process. An in-vitro study on phantom models showed 3D deviations of 1.89 mm at the implant apex, 1.35 mm at the implant shoulder and a mean angular deviation of 4.59º [27].

The prosthetic surgical template also offers less accuracy than a 3D-based surgical template, while only in two thirds of cases there is a relation to the actual bone situation [28]. Furthermore, the prosthetic surgical template is also less accurate than implant placement with navigation [29].

Navigation is another technique where mean deviations of 0.2–1.44 mm at the implant apex, 0.12–0.95 mm at the implant shoulder and a mean angular deviation of 1.35–4º were measured in several in-vitro studies [23], [27], [30]. Despite the accuracy and the potential benefit of image data-based navigation there is also the negative factor of cost-effectiveness, which makes it unsuitable for clinical routine [31], [32].

Finally, there are surgical templates available that are manufactured with CAD/CAM technology. In-vivo studies with tooth-supported templates resulted in deviations of 0.6–1.45 mm at the implant shoulder, 0.95–2.99 mm at the implant apex and angular deviations of 2.91–4.63º [33], [34]. The benefit of this technology is that no registration is needed and templates are manufactured based on 3D image data. These templates can be very useful especially in edentulous patients for producing bone-supported templates. CAD/CAM technology is already beginning to replace manual fabrication of surgical templates. 

## Conclusions

In conclusion, our study indicates very good transfer accuracy when using surgical templates for implant placement after 3D implant planning. The technique allows surgeons to protect important anatomical structures and facilitates implant positioning in relation to the intended superstructure so that the prosthetic restoration can be analyzed in advance. However, more clinical studies should be initiated to substantiate the promising results of the present study. 

## Notes

### Competing interests

The authors declare that they have no competing interests.

### Ethical considerations

All procedures performed in the study involving human participants were in accordance with the ethical standards of the institutional research committee and with the 1964 Helsinki declaration and its later amendments or comparable ethical standards. The study was approved by the ethics committee Ulm (Application Nr.: 311/09) and informed consent was obtained from all individual participants included in the study.

The publication is prepared in accordance to the STROBE Statement-Checklist. 

## Figures and Tables

**Table 1:**
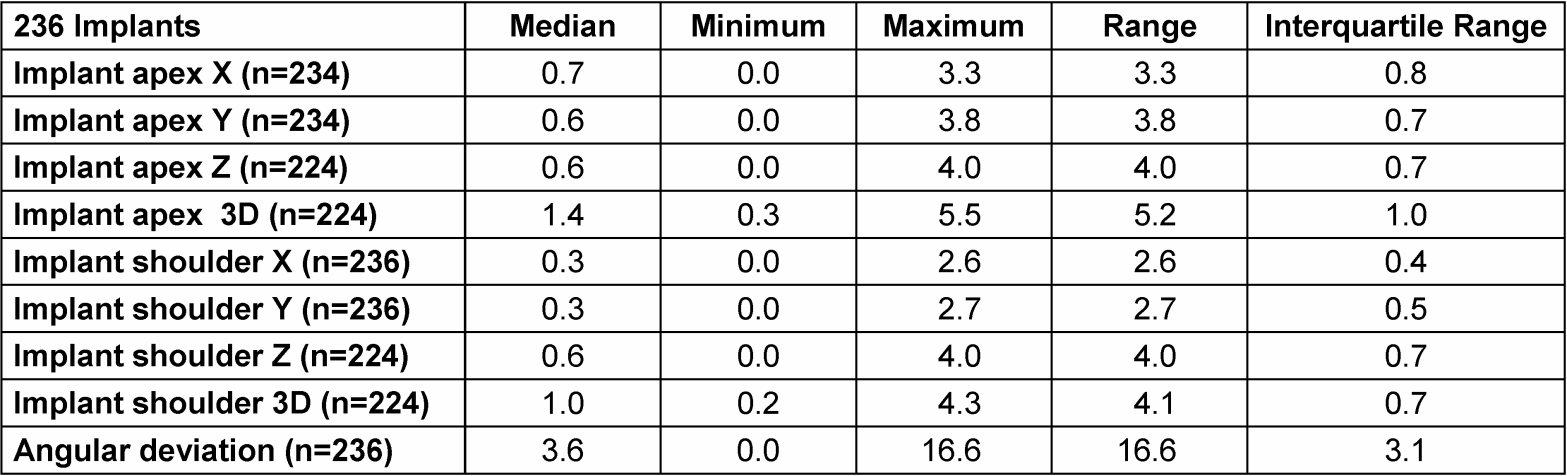
Deviations of 236 included implants in X-, Y-, Z-axis, 3D-deviation in mm and angular deviation in degrees at the implant shoulder and at the implant apex in consideration of the dropouts. n represents the included values for the statistical analysis (n=236 – dropout-values).
